# A chitosan-based antibacterial hydrogel with injectable and self-healing capabilities

**DOI:** 10.1007/s42995-023-00211-z

**Published:** 2023-12-20

**Authors:** Rui Chen, Yanan Hao, Secundo Francesco, Xiangzhao Mao, Wen-Can Huang

**Affiliations:** 1 State Key Laboratory of Marine Food Processing and Safety Control, College of Food Science and Engineering, Ocean University of China, Qingdao, 266404 China; 2 Laboratory for Marine Drugs and Bioproducts of Qingdao National Laboratory for Marine Science and Technology, Qingdao, 266237 China; 3 Qingdao Key Laboratory of Food Biotechnology, Qingdao, 266404 China; 4 Key Laboratory of Biological Processing of Aquatic Products, China National Light Industry, Qingdao, 266404 China; 5 Istituto di Scienze e Tecnologie Chimiche “Giulio Natta”, Consiglio Nazionale delle Ricerche via Mario Bianco 9, 20131 Milan, Italy

**Keywords:** Hydrogel, Chitosan, Silver ion, Cross-linking, Antibacterial

## Abstract

The presence of bacteria directly affects wound healing. Chitosan-based hydrogel biomaterials are a solution as they offer advantages for wound-healing applications due to their strong antimicrobial properties. Here, a double-cross-linking chitosan-based hydrogel with antibacterial, self-healing, and injectable properties is reported. Thiolated chitosan was successfully prepared, and the thiolated chitosan molecules were cross-linked by Ag–S coordination to form a supramolecular hydrogel. Subsequently, the amine groups in the thiolated chitosan covalently cross-linked with genipin to further promote hydrogel formation. In vitro experimental results indicate that hydrogel can release Ag^+^ over an extended time, achieving an antibacterial rate of over 99% against *Escherichia coli* and *Staphylococcus aureus*. Due to the reversible and dynamic feature of Ag–S coordination, an antibacterial hydrogel exhibited injectable and self-healing capabilities. Additionally, the hydrogel showed excellent biocompatibility and biodegradability.

**Supplementary Information:**

The online version contains supplementary material available at 10.1007/s42995-023-00211-z.

## Introduction

Bacterial infections are the main reason for an extended wound-healing period (Mohamed Salleh et al. 2022). While current dressings can ensure skin tissue regeneration, when wounds are associated with multidrug-resistant bacteria, traditional wound dressings are less frequently used (Gao et al. 2020; Kong et al. 2018). Hence, biomaterials with antimicrobial agents remain an urgent clinical need for chronic infectious wounds. Silver ion offers a promising alternative to conventional antimicrobial agents (Somayajula et al. 2019; Wu et al. 2009). It can attach to the cell surface, increase cytoplasmic membrane permeability, and rupture the bacterial envelope (Bapat et al. 2018; Morones et al. 2005; Xu et al. 2021). Silver ion also exhibits both strong and broad-spectrum antimicrobial activities but also regulate the inflammatory response, which can accelerate the wound-healing process (Alshameri and Owais 2022; Dai et al. 2016; Xu et al. 2021). A current limitation is that the antibacterial effect of silver ions can only be sustained for a short period, that is, until the silver ions are depleted. Incorporating silver ions into dressings is a proposed method to enhance and prolong antibacterial activity.

Chitosan (CS), a derivative of chitin, is commonly found in the exoskeletons of crustaceans (Rinaudo 2006). The key feature of chitosan is its antimicrobial capacity against a broad range of bacteria and fungi (No et al. 2002; Verlee et al. 2017). Due to these properties, chitosan has received a lot of attention in the field of biomedicine, having been used in various formulations, such as hydrogels, sponges, and bandages (Hamedi et al. 2018; Tchemtchoua et al. 2011; Wang et al. 2016). However, the chitosan-based materials usually have a low degree of chemical complexity and weak mechanical strength (Zhang et al. 2021). These limitations can be improved by chemical modification.

Here, we report a chitosan-based antibacterial hydrogel with injectable and self-healing capabilities for bacteria-associated wound healing. The hydrogel dressing was prepared via coordinative cross-linking of thiolated chitosan with silver ions that were further covalently cross-linked with genipin (GP). We investigated the chemical structure, morphology, biocompatibility, biodegradability, in vitro antibacterial properties, and rheological properties of the hydrogels.

## Materials and methods

### Materials

Chitosan (Avg. MW: 300 kDa; DD: 90–95%) and 1-hydroxybenzotrizole (HOBt) were purchased from Macklin Biochemical Co., Ltd, China. N-acetyl-l-cysteine (NAC), 1-ethyl-3-(3-dimethylaminopropyl)-carbodiimide hydrochloride (EDC·HCl), and 5,5-dithio-bis-(2-nitrobenzoic acid (DTNB, Ellman’s Reagent) were purchased from Yuanye Bio-Technology Co., Ltd, Shanghai, China. Genipin (GP) was purchased from Herbochem Biotech Co., Ltd. Dulbecco's Modified Eagle Medium (DMEM), 0.25% trypsin–EDTA, and phosphate-buffered saline (PBS) were obtained from Labgic (Biosharp) Technology Co., Ltd. Cell Counting Kit-8 (CCK-8) was purchased by Beyotime Biotechnology Co., Ltd. Other chemicals were obtained from Sinopharm Chemical Reagent Co. Ltd. All chemicals and solvents were used without further purification.

The bacterial strains of *Escherichia coli* (*E. coli*) and *Staphylococcus aureus* (*S. aureus*) and fibroblast cells (L929) were obtained from the China Center for Type Culture Collection (CCTCC). Luria–Bertani broth (LB broth) and Luria–Bertani agar (LB agar) were used in growing and maintaining the bacteria. DMEM was used for the cell culture.

### Synthesis of the thiolated chitosan

Chitosan-N-acetyl-l-cysteine (CS–NAC) was prepared by a previously reported method (Krauland et al. 2005; Wang et al. 2009). Briefly, CS (1.000 g) and HOBt (0.697 g, 5.16 mmol) were added into DI water (92.0 mL) and stirred until a transparent solution was formed. Next, NAC (3.368 g, 20.64 mmol) and EDC (7.913 g, 41.28 mmol, 8.0 mL) were mixed into the resulting solution successively. Then, pH of the solution was adjusted to 5.0 using 1 mol/L HCl, and the mixture was stirred for 3 h at room temperature. The resultant solution was dialyzed (Mw 8000–14,000 cut) against 5 mmol/L HCl containing 2 mmol/L EDTA at 4 ℃ for 2 days, then against the same medium with additional 1% NaCl for 3 days, and finally against 1 mmol/L HCl for 2 days in a dark space. The dialyzed solution was lyophilized at − 50 ℃ and stored at 4 ℃ in the dark condition for further use. The amount of free thiol groups on the CS-NAC was determined using Ellman’s reagent. The chemical structure of CS-NAC was characterized by Fourier transform infrared spectroscopy (FT-IR, 4000–400 cm^−1^, KBr pellets, Thermo Scientific Nicolet iS5) and proton nuclear magnetic resonance (^1^H NMR, D_2_O, TMS, ppm, 500 MHz, Bruker Avance III 500 M).

### Preparation of CS-NAC/Ag^+^/GP hydrogels

CS-NAC was dissolved in a 1% (v/v) acetate buffer solution (pH 5.0) while stirring at room temperature until a yellow solution (1.5%, m/v) was obtained. GP (0.3 and 0.6 mg/mL, 1.0 mL) and AgNO_3_ (0.6 and 6 mg/mL, 1.0 mL) were added to the CS-NAC solution (4.0 mL) while stirring. The mixtures were stored under dark conditions at 37 ℃ for 12 h and then lyophilized at – 50 ℃ to obtain porous hydrogels. The hydrogels were named CS-NAC/Ag^+^-*x*/GP-*y*, where *x* and *y* are the final concentrations of Ag^+^ and GP in the hydrogel system, respectively. Ellman’s method and ninhydrin assay were used to measure the concentrations of the unreacted thiol groups and amine groups, respectively (Ellman 1959; Rosen 1957). The cross-linking degree of CS-NAC/Ag^+^/GP hydrogels was measured.

### Characterization

The chemical structure of the prepared CS-NAC/Ag^+^/GP hydrogels was characterized by FT-IR (Thermo Scientific Nicolet iS5) in the range of 4000–400 cm^−1^ at a resolution of 0.5 cm^−1^. KBr pellets were formed after the hydrogels were thoroughly washed with deionized water and lyophilized. Featured elements were analyzed by X-ray photoelectron spectroscopy (XPS, Thermo Scientific ESCALAB 250Xi system) using 200 W monochromatic Al Kα radiation. The morphology of the hydrogels was examined using scanning electron microscopy (SEM, FEI quanta FEG 250) at 20 kV.

### Porosity of CS-NAC/Ag^+^/GP hydrogels

The porosity of the CS-NAC/Ag^+^/GP hydrogels was measured by a previously reported method (Liang et al. 2016). Each lyophilized hydrogel sample was immersed in absolute ethanol until saturated. The weight of the hydrogel was measured before and after immersion. The porosity (P) was calculated using the following equation:

$$ {\text{Porosity }}\,{\text{(\%) }}\, = \,\frac{m_2 - m_1 }{{\rho V}} \times 100. $$

where *m*_1_ is the initial weight of the hydrogel; *m*_2_ is the weight after immersing hydrogel into alcohol; *V* is the original volume of the hydrogel; and *ρ* is the density of alcohol.

### Rheological properties of hydrogels

The rheological properties of the hydrogels were analyzed using a rheometer (Anton Paar Physica MCR 301). Hydrogels were squeezed by a parallel plate geometry with the diameter of 50 mm and retained a gap of 1.0 mm. The shear storage modulus (*G*′) and shear loss modulus (*G*′′) were obtained at constant deformation (1.0%) with increasing frequency (from 1 to 20 Hz).

### Mechanical properties of hydrogels

The compression properties of CS-NAC/Ag^+^/GP hydrogels were calculated by compression measurement using a texture analyzer (SMS TA. XT Plus C). Briefly, cylindrical hydrogels were compressed at a rate of 0.1 mm/min. The process stopped when the compression strain was up to 70%. The stress–strain behavior of hydrogels was measured during tests.

### Self-healing and injectability of hydrogels

Hydrogels dyed with aniline blue or carmine were cut into two parts across their center line, respectively. The two parts were then placed in the original sealed mold with surface contact. After incubating for 6 h at 37 ℃, the hydrogel was stretched to determine the self-healing capability by observation. The healed hydrogels were mechanically measured at room temperature to study self-healing properties. In addition, the continuous injectable property of CS-NAC/Ag^+^-*1.00*/GP-*0.05* hydrogel was investigated using a 5-mL syringe equipped with a 0.7-mm-diameter needle.

### Swelling behavior

Hydrogels were immersed in a 10 mmol/L HCl solution (pH 2.0) and a 10 mmol/L PBS buffer (pH 7.4), respectively. The excess liquid on their surface was absorbed by filter paper and the swollen hydrogels weighed at predetermined time intervals (10–180 min). The swelling ratio of the hydrogels was calculated by the following formula (Chen et al. 2009):

$$ {\text{Swelling}}\,\,{\text{ratio }}(\% )\, = \, \frac{m_t - m_0 }{{m_0 }} \times 100. $$

where *m*_0_ is the weight of the original samples and *m*_t_ is the swollen weights at a specific time.

### In vitro antibacterial studies

The spread plate method was used to evaluate the antibacterial activity of the hydrogels (Sun and Sun 2002). *E. coli* (gram-negative) and *S. aureus* (gram-positive) were used as model bacteria. *E. coli* and *S. aureus* were inoculated in sterilized LB broth at 37 °C for 12 h while continuously shaking at 220 r/min. The bacterial suspensions were diluted to 1 × 10^6^ CFU/mL with PBS buffer (pH 7.4). Next, diluted bacterial dispersion and frozen dried hydrogels were added to 24-well culture plates and incubated at 37 °C for 2 h. The hydrogel-treated solutions were plated onto LB agar after being diluted with PBS buffer. Agar plates were placed for 12 h for *E. coli* and 18 h for *S. aureus* at 37 °C, respectively. The number of CFUs on the LB agar plates was counted to determine the antibacterial activity. The bacterial survival ratio was calculated using the following equation:

$$\text{Survival ratio }(\mathrm{\%}) = \frac{\text{CFU}}{{\text{CFU}}_{0}}\times 100.$$

where CFU is the number of colony-forming units after hydrogel treatment and CFU_0_ is the initial number of colony-forming units.

To examine the morphologies of the hydrogel-treated bacteria, bacteria were immersed in hydrogels for 2 h and fixed with 2.5% (v/v) glutaraldehyde for 1 h. Subsequently, hydrogels with bacteria were rinsed with sodium phosphate buffer and dehydrated with a graded ethanol series. SEM images were taken at an acceleration voltage of 20 kV.

### In vitro degradation test

The degradation of the hydrogels was determined by a previously reported method (Tran et al. 2011). The CS-NAC/Ag^+^/GP hydrogels were immersed in 50.0 mL of PBS solution (pH 5.6) at 37 °C for 16 days. The weight of the hydrogels was measured on days 1, 2, 3, 5, 8, 10, 12, and 16. Then, a fresh PBS solution was used to supplement the samples. The rheological properties (*G*' and *G*'') of the samples (days 0, 2, 5, and 10) were measured using a rheometer under a constant deformation of 1.0% and increasing frequency ranging from 1 to 20 Hz. The degradation of the hydrogels was calculated using the equation:

$$\text{Degradation }(\mathrm{\%}) = \frac{{\text{m}}_{0 }\text{- }{\text{m}}_{\text{m}}}{{\text{m}}_{0}} \times 100.$$

where *m*_0_ and *m*_m_ are the weights of the initial hydrogels and those immersed for a specific time, respectively.

### In vitro Ag^+^ release studies

The hydrogels were incubated in 50 mL of PBS solution (pH 5.6) at 37 °C for 24 days. At various time points, next, 5 mL of the solution was drawn and the concentration of released Ag^+^ was measured by inductively coupled plasma mass spectrometry (ICP-MS, Agilent 7700 s). Then, 5 mL of fresh PBS was added to the solution.

### In vitro cytotoxicity and cell proliferation tests

The cell cytotoxicity and proliferation were analyzed with an L929 fibroblast suspension via CCK-8 assay. The CS-NAC/Ag^+^/GP hydrogels were sterilized, and the hydrogels were immersed in the DMEM at 37 °C for one day. The resulting solutions were sterilized by filtration (filter diameter = 220 nm) with calf serum added to the resulting solution of 10% (v/v).

L929 cells (100 μL) were seeded onto a 96-well culture plate with 5000 cells per well and incubated for 12 h at 37 °C/5% CO_2_. Then, the initial media was discarded and replaced with 100 μL of hydrogel-conditioned media. Fresh DMEM with 10% (v/v) calf serum was used as a negative control. CCK-8 (10 μL) was added to each well after incubation on days 1, 2, and 3. Absorbance was measured with a microplate reader at a wavelength of 450 nm.

The hydrogels (CS-NAC/Ag^+^-*0*/GP-*0.05*, CS-NAC/Ag^+^-*1.00*/GP-*0*, and CS-NAC/Ag^+^-*1.00*/GP-*0.05*) in 12-well plates were sterilized by 75% (v/v) ethyl alcohol and immersed in fresh DMEM. Then, the cells (50,000 cells per well) were added to each well to attach to the hydrogels. Cells were evaluated by a live/dead assay after incubation on days 1, 2, and 3 using a confocal laser scanning microscopy (Nikon A1R HD25).

### Statistical analysis

Results are presented as mean ± standard deviation (SD). The statistical analysis was conducted using one-way analysis of variance (ANOVA). Significance differences are shown with *P < 0.05, **P < 0.01.

## Results

### Synthesis of CS-NAC molecule and preparation of CS-NAC/Ag^+^/GP hydrogels

**Fig. 1:**
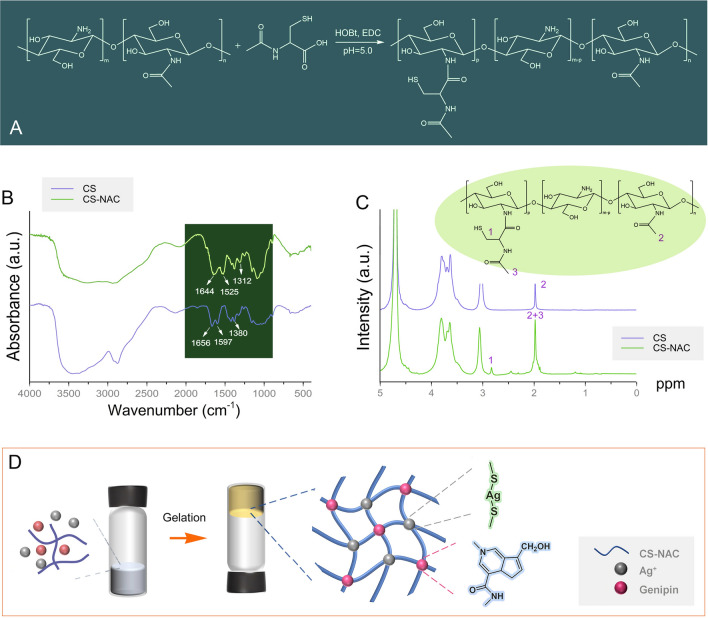
**A** Synthesis of CS-NAC; **B** FT-IR spectra of CS-NAC and chitosan; **C** ^1^H NMR spectra of CS-NAC and chitosan; and **D** schematic architecture of CS-NAC/Ag^+^/GP hydrogels

Synthesis of CS-NAC is shown in Fig. 1A. The thiol group content on CS-NAC was determined to be 292.32 ± 5.98 μmol/g CS using Ellman’s method. The structure of the CS-NAC was confirmed by FT-IR and ^1^H NMR. FT-IR results showed that absorption peaks of the amide bond at 1644 cm^−1^, 1525 cm^−1^, and 1312 cm^−1^ were stronger than those of the CS, indicating successful modification from the conjugation of the NAC (Wang et al. 2009). As shown in Fig. 1C, the peak at 2.82 ppm was assigned to the—CH_2_SH of the NAC. The peak for the N-acetyl methyl protons at 1.98 ppm was enhanced compared with the spectrum of CS as a result of conjugation.

CS-NAC/Ag^+^/GP hydrogel was obtained by Ag–S coordination and genipin-mediated cross-linking of CS-NAC (Fig. 1D). Free thiol groups of the CS-NAC formed strong covalent bonds with Ag^+^ (Ag–S bond). The free amino groups were cross-linked by GP. To assess the number of covalent bonds, the free thiol groups and amino groups of the hydrogels were determined via Ellman’s method and ninhydrin assay, respectively. Only 6 mol% of the thiol groups were oxidized to disulfide bonds before the hydrogels were prepared. The number of disulfide bonds was not sufficient for gelation (Supplementary Figs. S1, S2). The content of the free thiol groups in the hydrogels showed that there were more Ag–S bonds with more Ag^+^, while the cross-linking degree by GP remained constant (Supplementary Fig. S2). Meanwhile, the increase in GP concentration increased the amine consumption during cross-linking up to the maximum cross-linking degree (35%), while the cross-linking degree by Ag^+^ remained constant. Results show that cross-linking by Ag^+^ and GP did not affect each other during hydrogel formation.

**Fig. 2:**
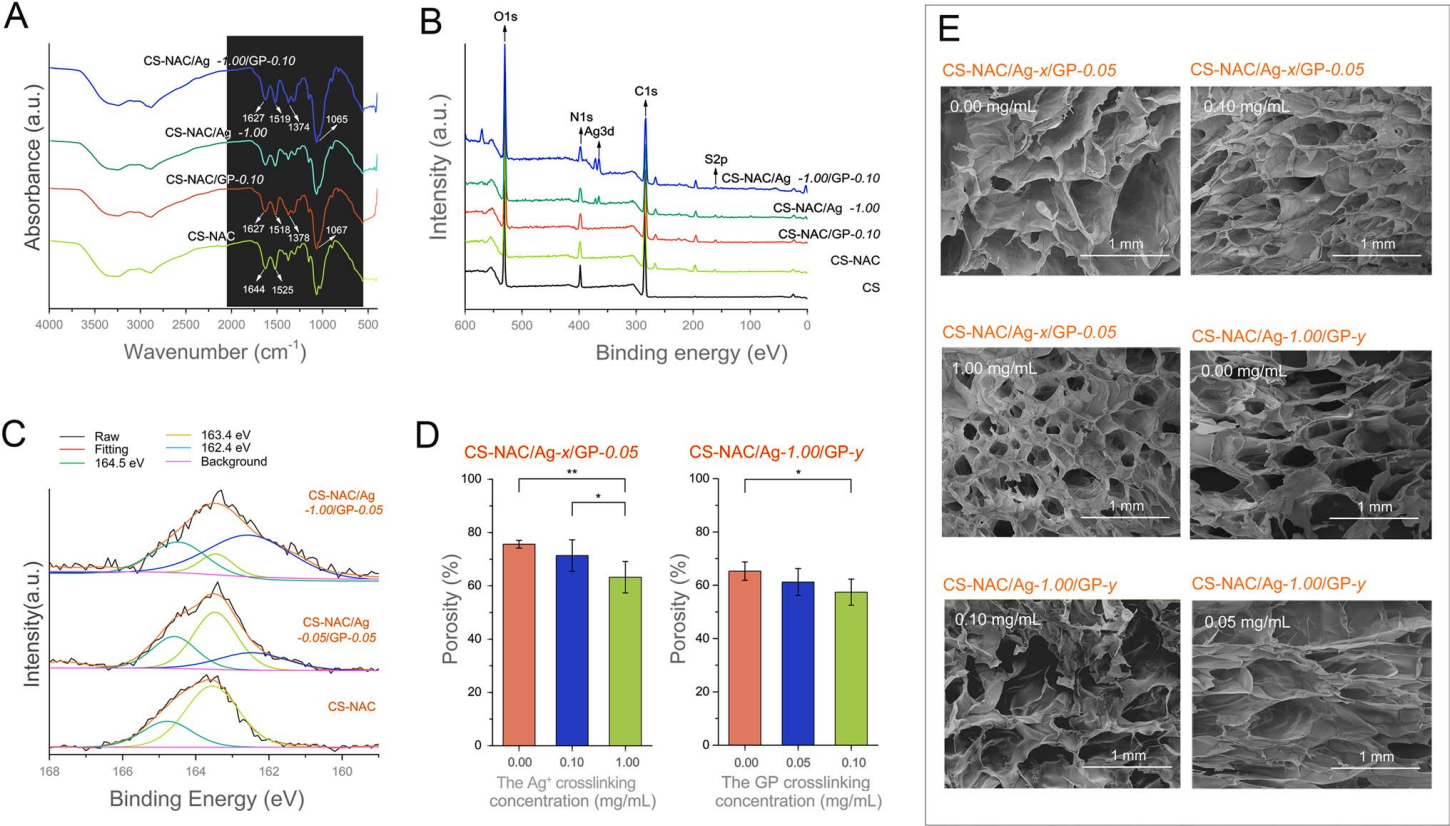
Characterization of CS-NAC/Ag^+^/GP hydrogels with different Ag^+^ and genipin concentrations. **A** FT-IR spectra, **B** XPS patterns, **C** XPS S2p spectra, **D** porosity, and **E** SEM images of the hydrogels (*n* = 3, mean ± SD, *P < 0.05 and **P < 0.01)

The characterization of the CS-NAC/Ag^+^/GP hydrogels compared to control groups is presented in Fig. 2. The FT-IR spectra demonstrated that the absorption of the secondary amide groups was reduced to 1627 cm^−1^ and 1518 cm^−1^, respectively, after cross-linking with GP. This can be attributed to nucleophilic substitution of the ester group on GP by the primary amine group on CS-NAC and the formation of secondary amides under an acid condition (Klein et al. 2016). Additionally, the increase shown in the peaks around 1378 cm^−1^ and 1067 cm^−1^ after cross-linking with GP can be attributed to absorption from C–N stretching vibrations and C–OH stretching vibrations, respectively. The XPS results exhibited the typical peaks of S2p, Ag3d, C1s, O1s, and N1s species (Fig. 2B). Compared with the spectrum of CS-NAC, the S2p spectra of hydrogels CS-NAC/Ag^+^-*0.05*/GP-*0.05* and CS-NAC/Ag^+^-*1.00*/GP-*0.05* were divided into the peaks of 162.4, 163.4 and 164.5 eV. Three peaks were corresponding to Ag–S bonds between CS-NAC and Ag^+^, thiol groups of CS-NAC unbounded to Ag^+^, and S–C bonds of CS-NAC, respectively (Pan et al. 2018). When Ag^+^ increased from 0.05 to 1.00 mg/mL, the intensity of the peak at 162.4 eV gradually increased, which demonstrated that higher amounts of thiol groups in CS-NAC were covalently linked to Ag^+^.

Micromorphology of the hydrogels was confirmed by SEM. As shown in Fig. 2E, although all hydrogels exhibited porous structure, the porosity was lower in the hydrogels with higher Ag^+^ and GP concentrations. The decreased porosity of the structure can be attributed to the cross-linking via Ag–S and NH_2_-GP, which reduced the space between the polymer chains (Fig. 2D).

### Rheological and mechanical properties of hydrogels

**Fig. 3:**
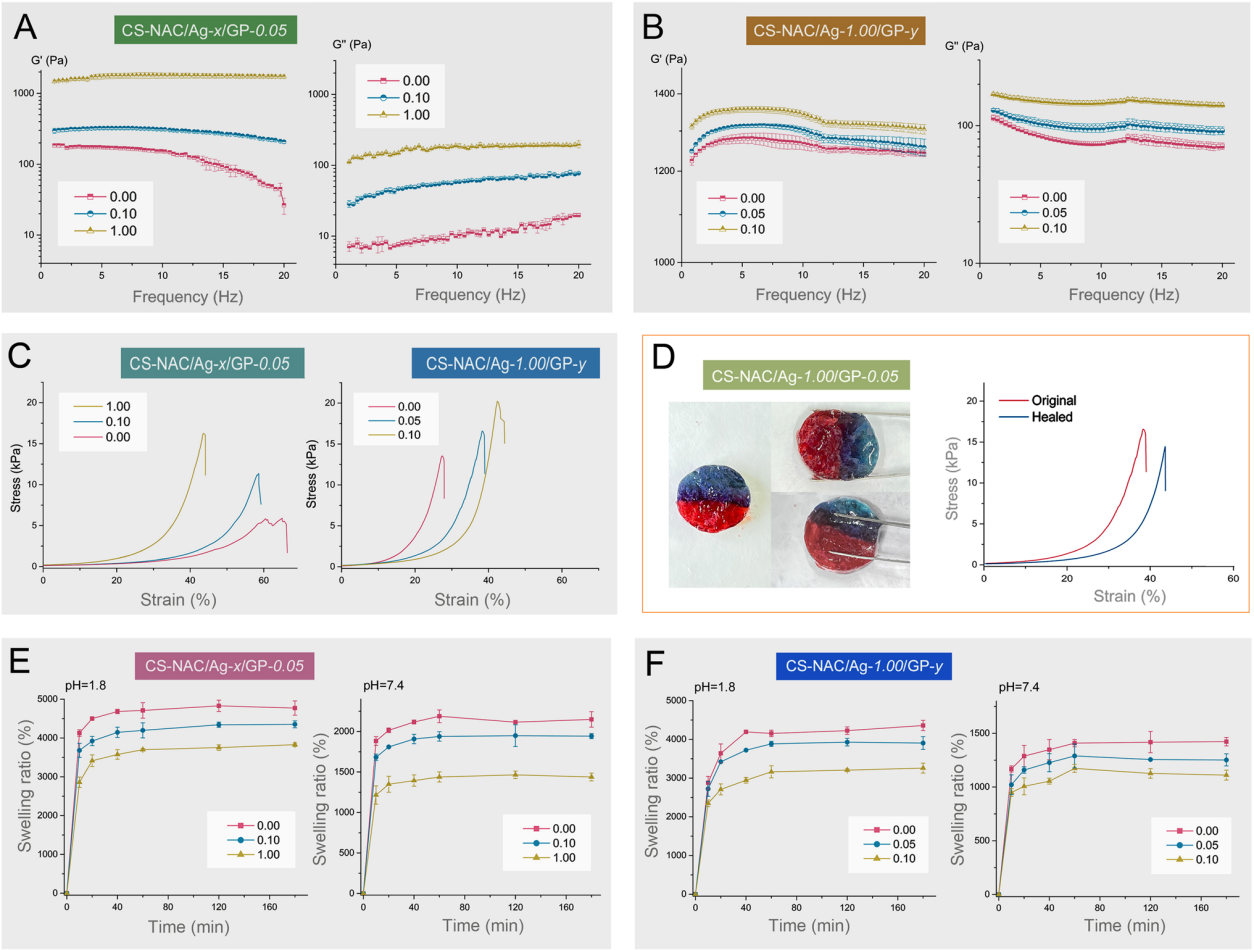
**A** Frequency sweeps (*G*′ and *G*″) of the CS-NAC/Ag^+^/GP hydrogels with different Ag^+^ concentrations (1 mm gap, *n* = 3, mean ± SD); **B** frequency sweeps of the hydrogels with various concentrations of genipin (*n* = 3, mean ± SD); **C** the compressive stress–strain curves of the hydrogels with different concentrations of Ag^+^ and genipin; **D** photographs of healing processes and compressive stress–strain curves of before and after wound healing; and **E** swelling ratio of the CS-NAC/Ag^+^/GP hydrogels (*n* = 3, mean ± SD)

Both G′ and G′′ present as a function of frequency (*ω*) at a strain (*γ*) of 1.0%. The hydrogels retain viscoelastic property at this strain via strain sweep experiments (Supplementary Fig. S4). Figures 3A and B show the rheological properties of the CS-NAC/Ag^+^/GP hydrogels. All hydrogel states were confirmed with G′ > G″ at all frequencies, which is a typical characteristic of hydrogels. As shown in Figs. 3A and B, higher G′ and G'' values were obtained at higher concentrations of Ag^+^ and GP.

The mechanical properties of the hydrogels with various cross-linker concentrations were measured by a compressive test. As shown in Fig. 3C, the CS-NAC/Ag^+^-*1.00*/GP-*0.05* hydrogel broke at stresses of 16.35 kPa, which was 1.94 and 0.44 times higher than the ultimate stress of CS-NAC/Ag^+^-*0*/GP-*0.05* and CS-NAC/Ag^+^-*0.10*/GP-*0.05* hydrogels, respectively. Furthermore, the ultimate stress and strain of hydrogels were increased with various GP concentrations, consistent with the rheological analysis.

### Self-healing property and injectability of hydrogels

The self-healing and injectable capabilities of hydrogels at physiological conditions are critically important for use in wound dressing. The CS-NAC/Ag^+^-*1.00*/GP-*0.05* hydrogel was stained into different colors to show the interface. After contacted for 6 h, the two half pieces self-healed into a whole hydrogel without any visible cracks, as shown in Fig. 3D. Self-healing performance was also confirmed by a compression test, demonstrating its self-healing efficiency of 88.36 ± 2.34%. The self-healing behavior of the hydrogel is attributed to the network in formation of the dynamic Ag–S bonds. The rebinding of covalent bonds occurred in the hydrogel network, endowing the hydrogel with self-healing ability. Moreover, the hydrogel could be injected through a syringe with a 0.7-mm needle maintaining the shape of the letters “OUC,” confirming continuous injectability of the hydrogel (Supplementary Fig. S5).

### Swelling behavior of hydrogels

Figures 3E and F present the swelling behavior of the various hydrogels. The dried hydrogels can quickly absorb a large amount of solution within 10 min in all groups. Moreover, the swelling behavior was closely related to the pH value. After 3 h, at pH of 2.0 and 7.4, the swelling ratios of the CS-NAC/Ag^+^/GP hydrogels ranged from 3,900–4,800% and 1,400–2,100%, respectively. This can be assigned to the protonation of the amino groups on the CS-NAC at an acidic condition (pH 2.0) and deprotonation at a basic solution (pH 7.4). The protonation of the amino groups on the CS leads to chain relaxation and solvent diffusion, while deprotonation results in the shrinkage of the hydrogel (Zeng and Fang 2004). Notably, the pH responsiveness of the hydrogels cross-linked with GP was not obvious because of the lower amount of residual amino groups in the hydrogels. The rapid swelling ability and high equilibrium swelling ratio suggest that the hydrogel is appropriate for application in wound dressings.

### In vitro antibacterial activity of hydrogels

**Fig. 4:**
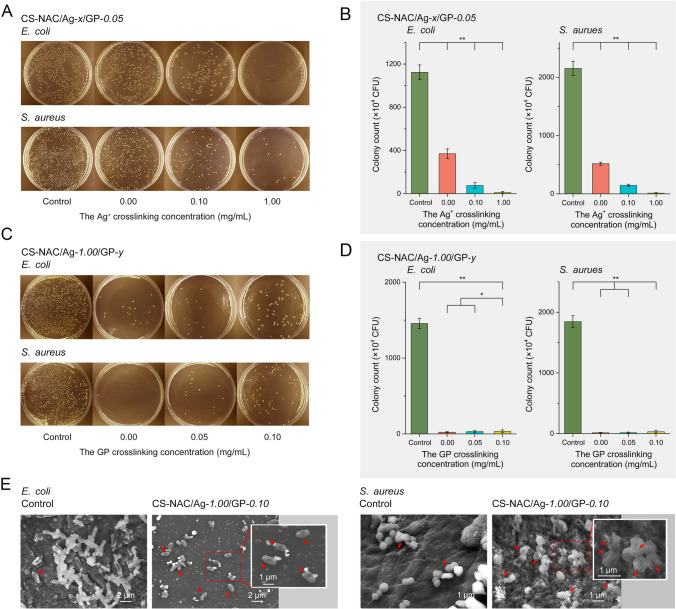
In vitro antibacterial activity of the hydrogels cross-linked with various Ag^+^ or genipin concentrations against *E. coli* and *S. aureus*. **A** and **C** Photographs of agar plates and **B** and **D** corresponding statistical data of the colonies of *E. coli* and *S. aureus*. (*n* = 6, mean ± SD, *P < 0.05 and **P < 0.01). **E** SEM images showing the morphological changes of *E. coli* and *S. aureus* after incubation of the CS-NAC/Ag^+^*-1.00*/GP*-0.05* hydrogel

The in vitro antibacterial ability of the hydrogels was tested against *E. coli* and *S. aureus*. As shown in Figs. 4A and B, the CS-NAC/Ag^+^-*0*/GP-*0.05* hydrogel had a survival ratio of 24.2 ± 2.6% for *S. aureus* and 32.9 ± 6.0% for *E. coli*. The antibacterial property of the hydrogel without Ag^+^ is attributed to the amine groups (–NH_2_) of the CS (Wu et al. 2014). The hydrogels with Ag^+^ exhibited a higher antibacterial activity compared with that of the hydrogel without Ag^+^. Antibacterial activity improved with an increasing concentration of Ag^+^. However, increasing the concentration of GP resulted in a decreased antibacterial activity (Fig. 4C, D). The results of the disk diffusion assay also confirmed the antibacterial activity of the hydrogels enhanced with increasing Ag^+^ content (Supplementary Fig. S6). These results revealed that antibacterial activities of the hydrogels can be derived from both CS and Ag^+^.

To determine the antibacterial process of the CS-NAC/Ag^+^-*1.00*/GP-*0.05* hydrogel, the morphology of the bacteria on the hydrogels was confirmed by SEM (Fig. 4E). When the CS-NAC/Ag^+^-*0*/GP-*0.05* hydrogel was applied, the bacteria maintained their original morphology. However, after contact with the CS-NAC/Ag^+^-*1.00*/GP-*0.05* hydrogel, significant morphological changes were observed in both bacterial strains. The surface of the bacteria was wrinkled and distorted. Shrunken *E. coli* and condensed *S. aureus* were also observed.

### In vitro release of Ag^+^

**Fig. 5:**
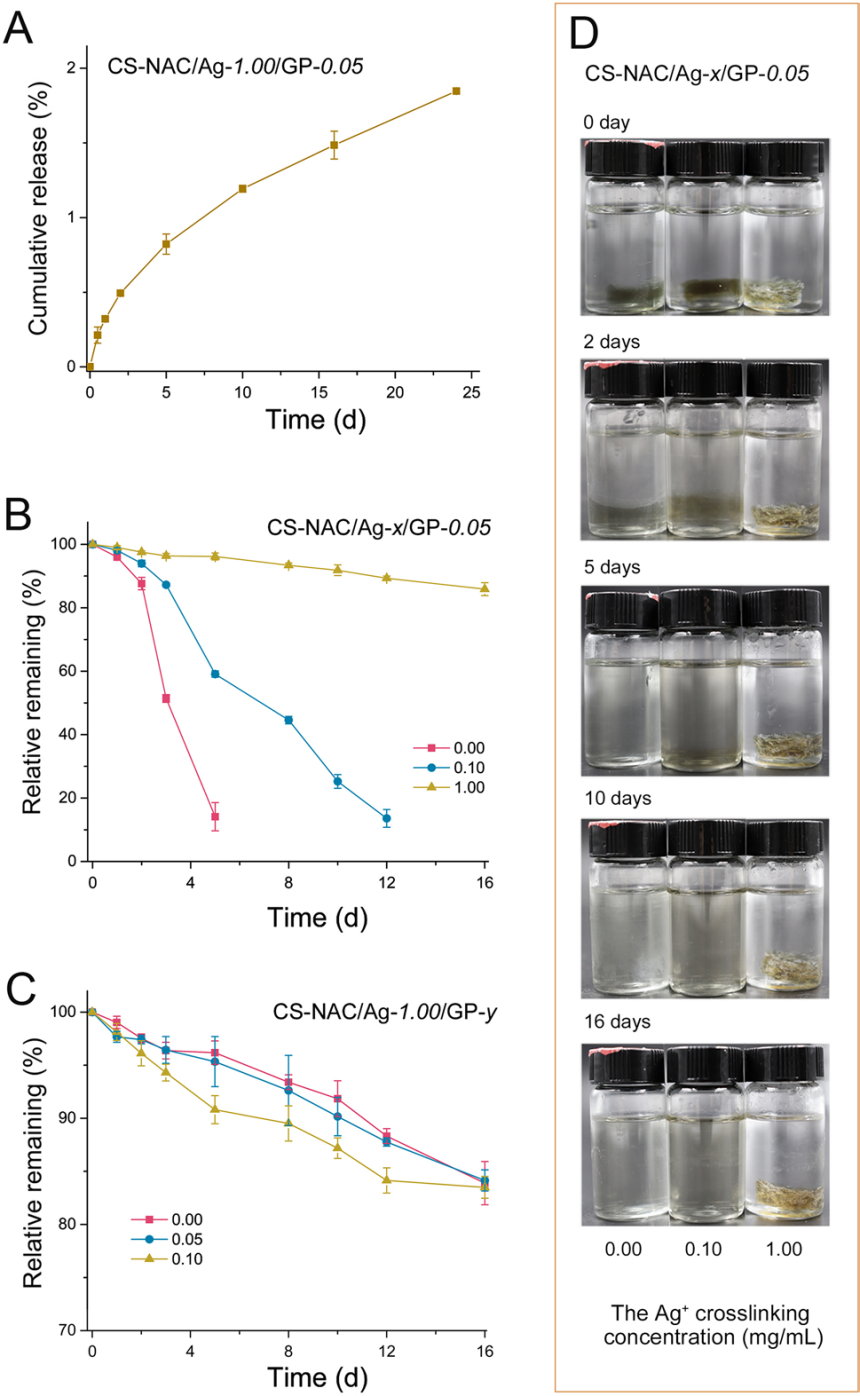
**A** Cumulative release profiles of Ag^+^ from the CS-NAC/Ag^+^-*1.00*/GP-*0.05* hydrogel (*n* = 3, mean ± SD). **B** Degradation behavior of the hydrogels with different Ag^+^ concentrations (*n* = 4, mean ± SD). **C** Degradation behavior of the hydrogels with various concentrations of GP. **D** Photographs of the degraded hydrogels

The release profile of Ag^+^ in the CS-NAC/Ag^+^-*1.00*/GP-*0.05* hydrogel was determined. The pH of the wound site is slightly acidic during the wound-healing period. The release behavior was evaluated at a pH of 5.6. The release (%) was calculated at specific time points. As exhibited in Fig. 5A, Ag^+^ was released continuously from the hydrogel over a long period of time.

### In vitro degradation of hydrogels

As shown in Fig. 5B, the CS-NAC/Ag^+^-*0*/GP-*0.05* hydrogel exhibited the most weight loss. In addition, G′ was lower than G″ on the fifth day of degradation, indicating the integrity of the hydrogel collapsed on day 5 (Supplementary Fig. S7). Increasing Ag^+^ content can slow the degradation rate of the hydrogels. The hydrogel structure collapsed on day 10 with the Ag^+^ concentration of 0.10 mg/mL, while the structure of hydrogel formed from the Ag^+^ concentration of 1.00 mg/mL remained intact for over 2 weeks (Fig. 5D). Meanwhile, the degradation of the hydrogels with various GP concentrations and a constant Ag^+^ concentration (1.00 mg/mL) did not show any significant changes. Approximately 85% of the weight of the hydrogels remained after 16 days (Fig. 5B). These results suggested that the overall structural integrity of the hydrogels was mostly maintained by Ag^+^. This occurs because imines can be hydrolyzed in aqueous solution, resulting in breaking of genipin-mediated cross-linking.

### Biocompatibility of hydrogels

**Fig. 6:**
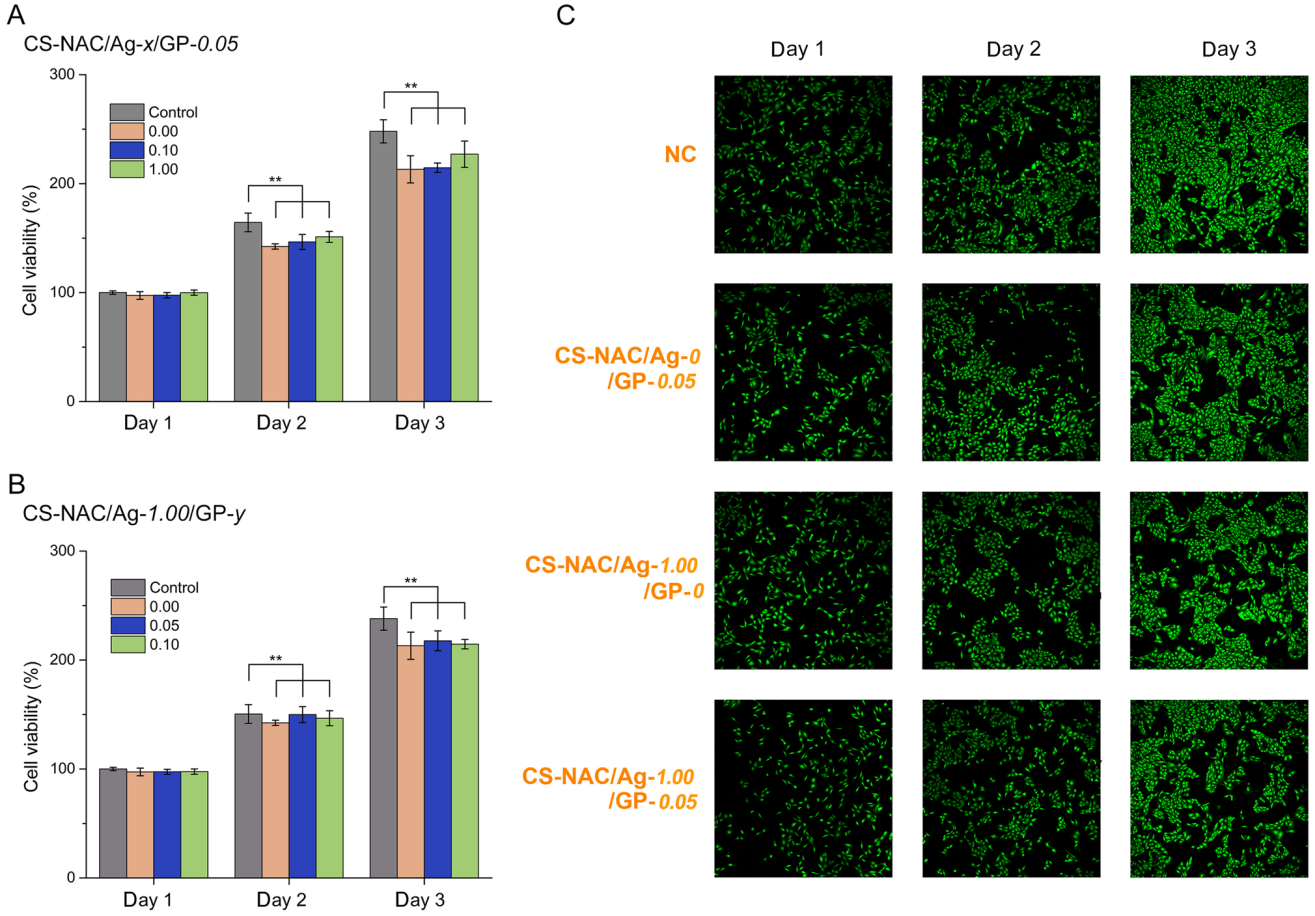
Biocompatibility analysis. **A**, **B** Cell viability of fibroblast cells cultured in the hydrogel-conditioned media. **C** Live/dead staining fluorescent images (*n* = 4, mean ± SD, *P < 0.05 and **P < 0.01)

The CCK-8 assay and live/dead assay were used to evaluate the cytocompatibility of the hydrogels. No observable cytotoxicity was detected in response to the hydrogel-conditioned solutions (Fig. 6A, B). Moreover, as presented in Fig. 6C, most cells were alive and the number of live cells increased as culture time increased, demonstrating that the hydrogels were cytocompatible. These results suggested that hydrogels have outstanding biocompatibility without observable negative effects on cell growth.

## Discussion

Chitosan can be cross-linked with different polymers or monomers to create biomaterials with great mechanical strength, ideal swelling, and antibacterial properties. Herein, CS-NAC/Ag^+^/GP hydrogels were obtained by Ag–S coordination and GP-mediated cross-linking of CS-NAC. The improvement of mechanical properties can be related to an increase in covalent bonds with high concentration of Ag^+^ and GP. The density of the network in hydrogels was improved with the increased degree of cross-linking, thus increasing the force required to destroy the hydrogel structure. The dynamic Ag–S coordination not only led to significant enhancements in the mechanical properties of the chitosan-based hydrogels but also improved antibacterial activity. However, the antibacterial activities of the hydrogels slightly decreased with an increased GP concentration, which can be attributed to the consumption of amine groups in the cross-linking by GP. Therefore, the CS-NAC/Ag^+^-*1.00*/GP-*0.05* hydrogel had enhanced antibacterial activity, achieving an antibacterial rate of over 99% against *E. coli* and *S. aureus* when Ag^+^ concentration was increased to 1.0 mg/mL The antibacterial process of this hydrogel is consistent with the antibacterial mechanism of Ag^+^ in that Ag^+^ can destroy the bacterial wall and membrane (Li et al. 2017). Moreover, self-healing and injectable capabilities are especially appealing for skin wound healing because these properties can help maintain the structure of dressings under external mechanical stress and also integrate ruptured dressings even after mechanical destruction. The hydrogel exhibited antibacterial, injectable, and self-healing capabilities because of Ag–S covalent bonds, showing great potential as a wound dressing caused by bacterial infections.

## Conclusions

In summary, we present a chitosan-based hydrogel dressing for bacteria-associated wound healing. In the presence of dynamic Ag–S coordination, this hydrogel composite exhibits excellent antibacterial activity, self-healing property, and injectable capability. We anticipate the hydrogel composite has great potential in the different types of wounds exposed to external mechanical stress with risks of bacterial infection.

### Supplementary Information

Below is the link to the electronic supplementary material.

Supplementary file1 (DOCX 19510 KB)

## Data Availability

The data that support the findings of this study are available from the corresponding author on reasonable request.
