# Supplementary material for: A chitosan-based antibacterial hydrogel with injectable and self-healing capabilities
Source: Mar Life Sci Technol. 2023 Dec 20;6(1):115–25. doi: 10.1007/s42995-023-00211-z (PMC10902234; doi:10.1007/s42995-023-00211-z)
Supplement: Supplementary file 1 — Supplementary file1 (DOCX 19510 KB) [file 42995_2023_211_MOESM1_ESM.docx]

**A chitosan-based antibacterial hydrogel with injectable and self-healing capabilities**

Rui Chen^a, c, d^, Yanan Hao^a, c, d^, Secundo Francesco^e^, Xiangzhao Mao^a, b, c, d^, Wen-Can Huang^a, c, d^ *

^a^ State Key Laboratory of Marine Food Processing and Safety Control, College of Food Science and Engineering, Ocean University of China, Qingdao 266404, China

^b^ Laboratory for Marine Drugs and Bioproducts of Qingdao National Laboratory for Marine Science and Technology, Qingdao 266237, China

^c^ Qingdao Key Laboratory of Food Biotechnology, Qingdao 266404, China

^d^ Key Laboratory of Biological Processing of Aquatic Products, China National Light Industry, Qingdao 266404, China

^e^ Istituto di Scienze e Tecnologie Chimiche "Giulio Natta", Consiglio Nazionale delle Ricerche via Mario Bianco 9, 20131, Milan, Italy

**Experimental Section**

**Determination of the Number of Free Thiols**

Ellman's test was performed using a standard procedure reported in the literature with some modifications (Wang et al. 2009). Briefly, thiol modified chitosan (1.0 mg) was dispersed in water (500 μL). The dispersed solution was added to phosphate buffer (500 μl, 0.2 mol/L, pH = 8.0) and DTNB (1.0 mL, 0.3 mg/mL). After a 2 h incubation at 25℃, the optical absorbance was measured at 412 nm. A calibration curve was carried out using L-cysteine as the standard.

**Crosslinking Degree by Genipin**

The ninhydrin method was used to determine the crosslinking degree of genipin, especially the amine groups of the samples. A D-glucosamine hydrochloride solution of 100 μmol/L in 20 mmol/L phosphate buffer (PB, pH = 5.6) was used to prepare the standard curve. Frozen dried hydrogel samples (1 mg) from each group were swollen for 12 h in 0.20 ml of 20 mmol/L PB (pH = 5.6). Ninhydrin reagent (0.4 mL) was added to the hydrogel mixture and heated to 100 °C in a water bath for 20 min. After cooled to room temperature, the solution was then centrifuged at 10000 g for 20 min and diluted 10 times using 95% ethanol. The absorbance of the solutions was recorded at 570 nm.

**UV-Vis Absorption Spectra of the Hydrogels**

The UV-vis spectroscopic measurements were carried out using a UV spectrometer (Shimadzu UV-2550, China) from 200 to 800 nm.

**Rheological Properties (strain factor)**

The rheological properties of the prepared hydrogel systems were determined with a parallel plate geometry (50 mm diameter; 1 mm gap). Shear storage and shear loss moduli (G′ and G′′, respectively) were obtained at a constant frequency (0.1 Hz) with an increasing strain (from 0.01 to 100%).

**In Vitro Antibacterial Activities of the Hydrogels**

*E. coli* and *S. aureus* were used to evaluate the antibacterial activity of the hydrogels. Thus, 100 μL of a 10^6^ CFU/mL bacteria suspension was spread onto an LB agar plate, and then, hydrogels 1.0 cm in diameter and 0.2 cm thick (sterilized by UV light) were placed onto the surface of the agar. After a 12 h incubation at 37 °C, the diameter of the inhibition zone was measured.

**Reference**

Wang X, Zheng C, Wu Z, Teng D, Zhang X, Wang Z, Li C (2009) Chitosan-NAC nanoparticles as a vehicle for nasal absorption enhancement of insulin. J Biomed Mater Res B Appl Biomater 88B:150-161.

**Results**

**
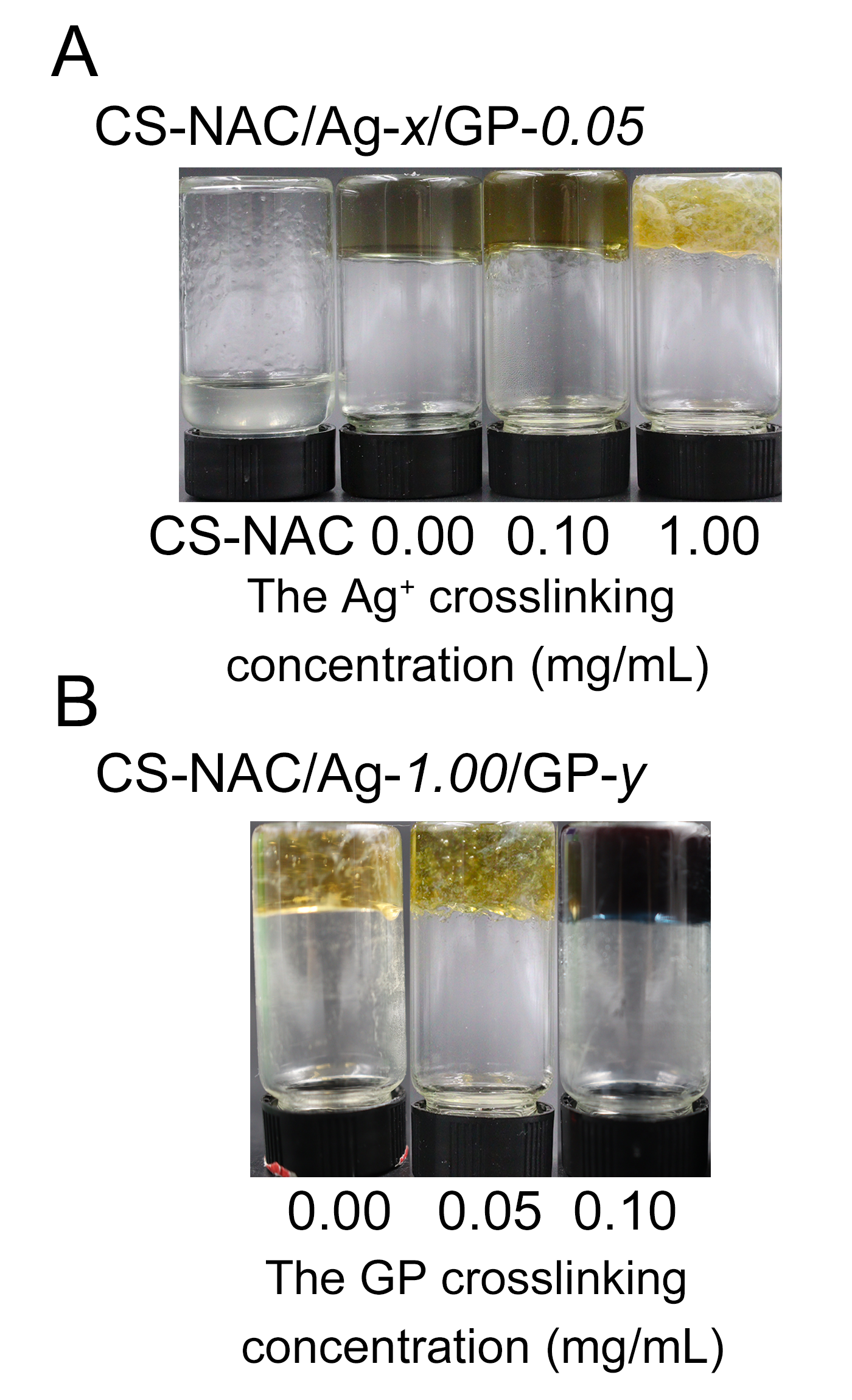
**

**Figure S1** Photograph of the hydrogels with different concentrations using a vial invert method


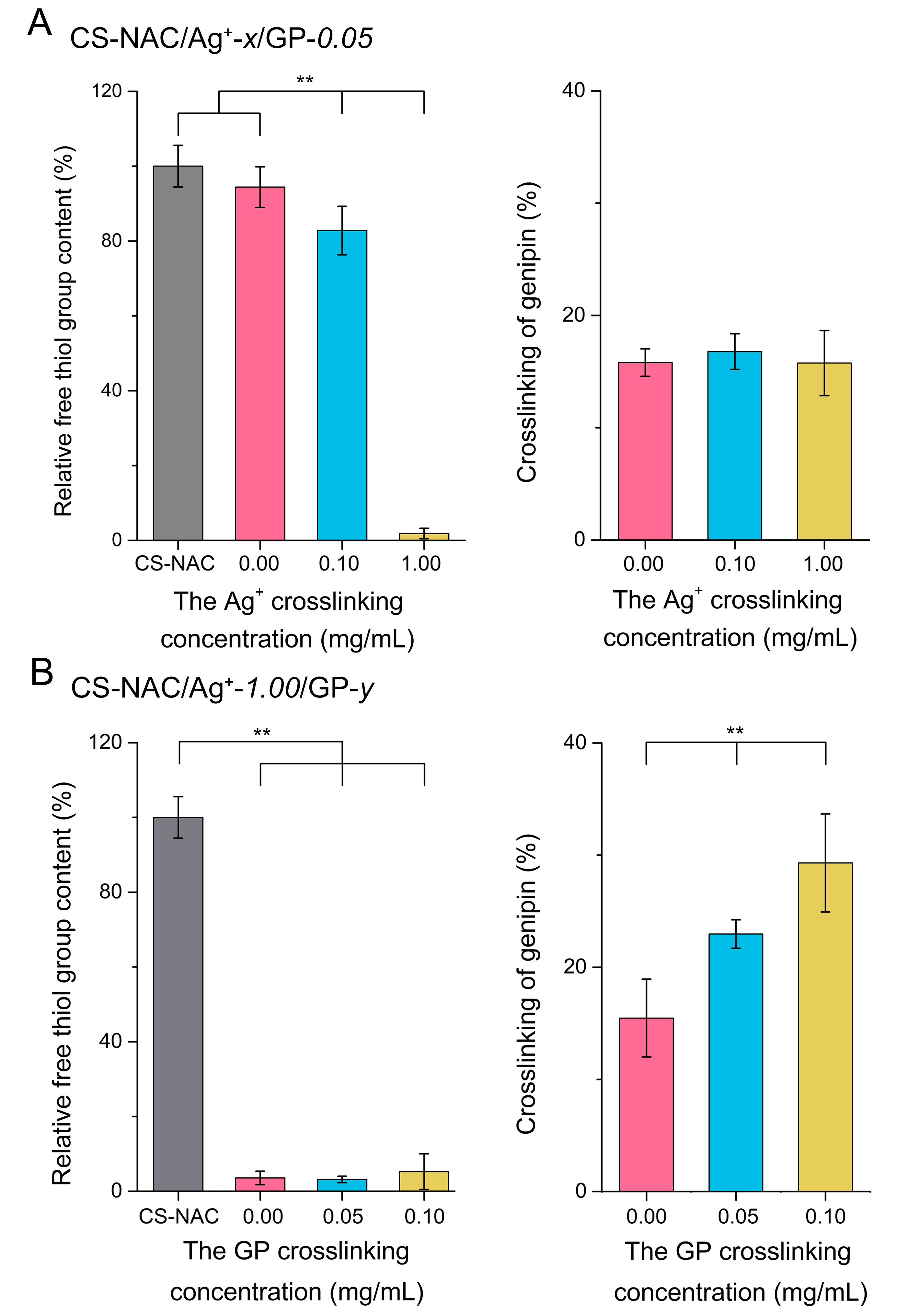


**Figure S2** Crosslinking degree of (A) the CS-NAC/Ag^+^/GP-*0.05* hydrogel, and (B) the CS-NAC/Ag^+^-1.00/GP hydrogel (n = 3, mean ± SD, **P* < 0.05 and ***P* < 0.01).


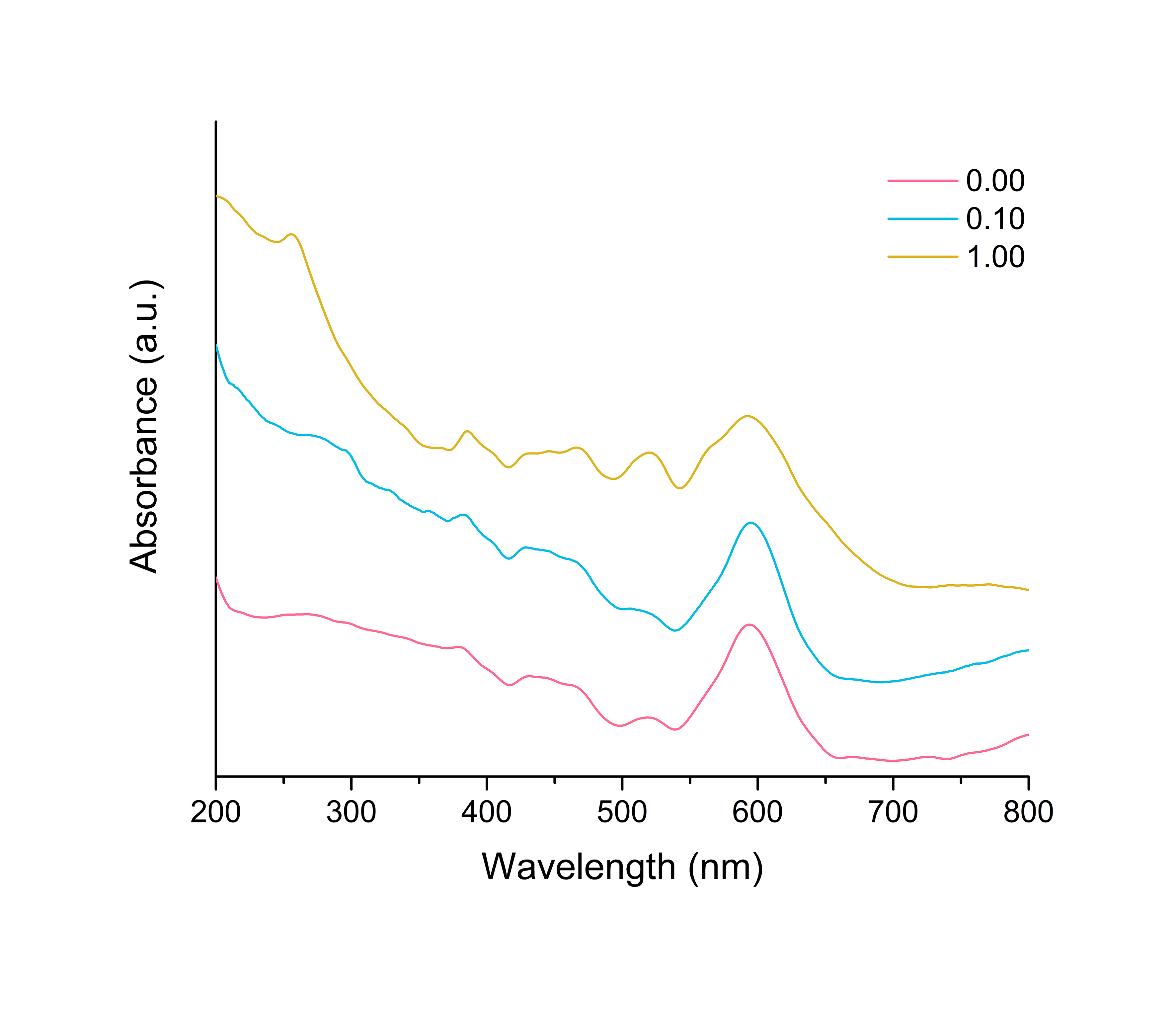


**Figure S3** UV-Vis absorption spectra of the CS-NAC/Ag^+^-*x*/GP*-0.05* hydrogels


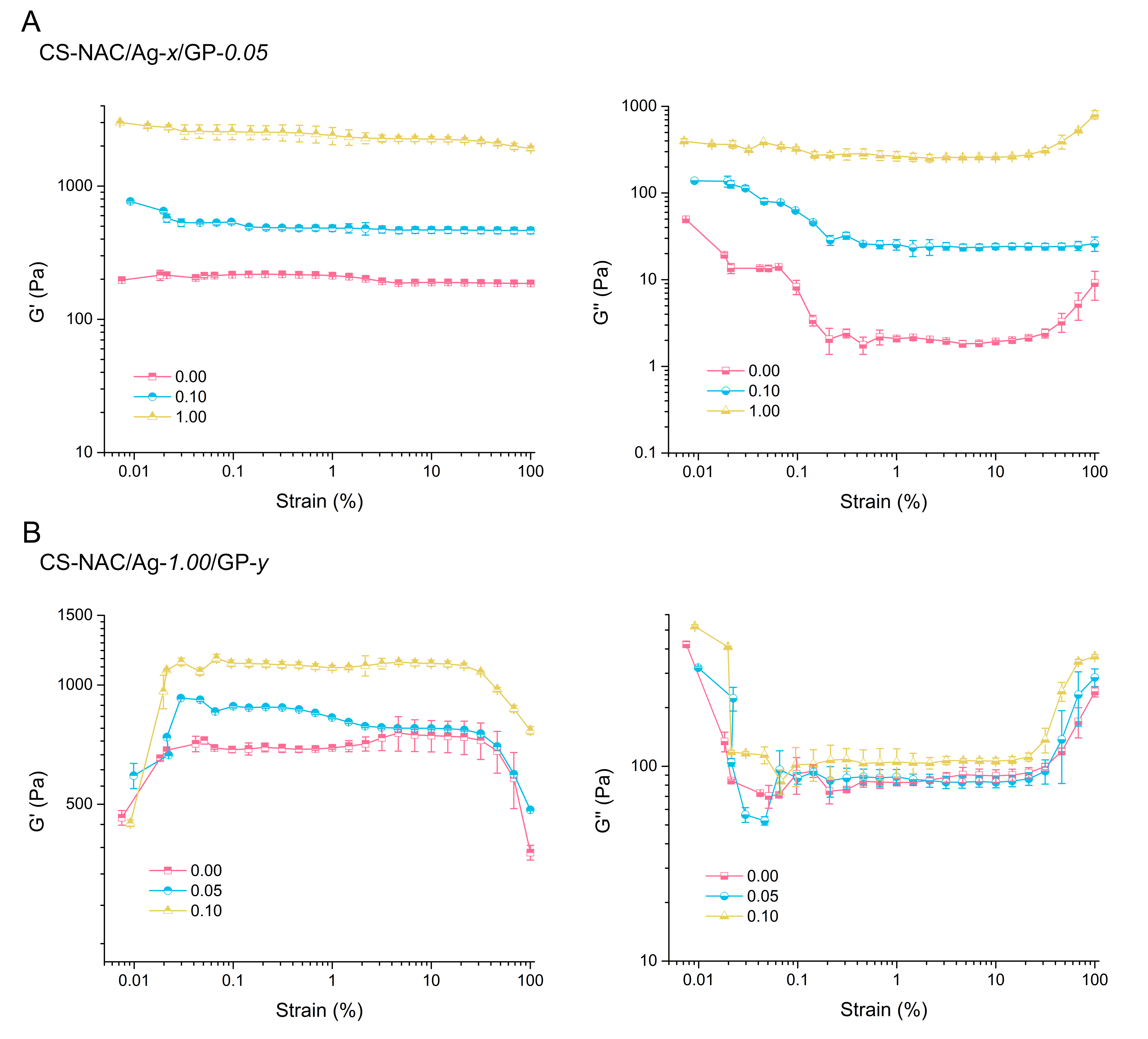


**Figure S4** Strain sweep of (A) the CS-NAC/Ag^+^/GP*-0.05* hydrogels and (B) the CS-NAC/Ag^+^*-1.00*/GP hydrogels at constant frequencies (0.1 Hz).


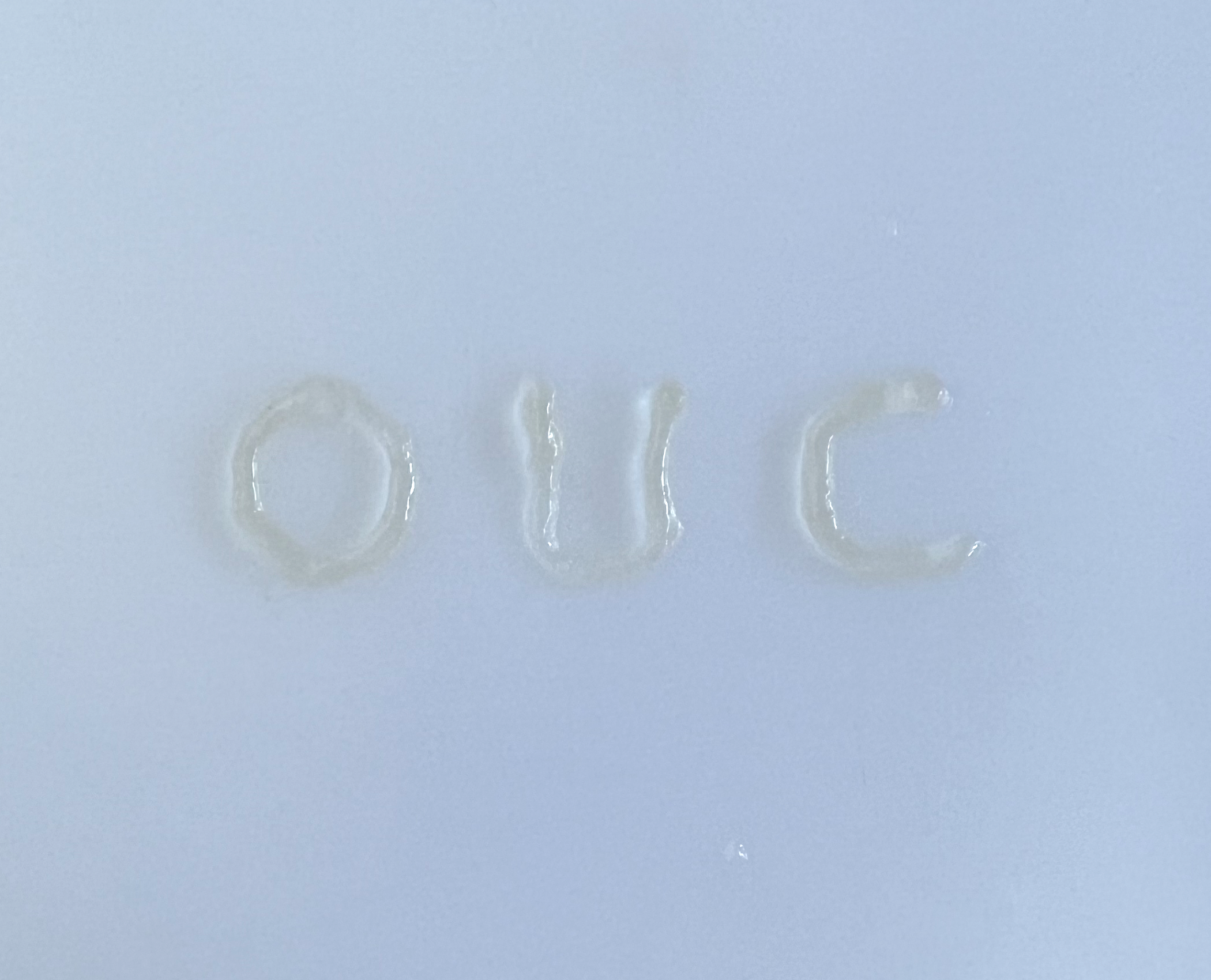


**Figure S5** The injectable property of CS-NAC/Ag^+^*-1.00*/GP-*0.05* hydrogel*.*

**
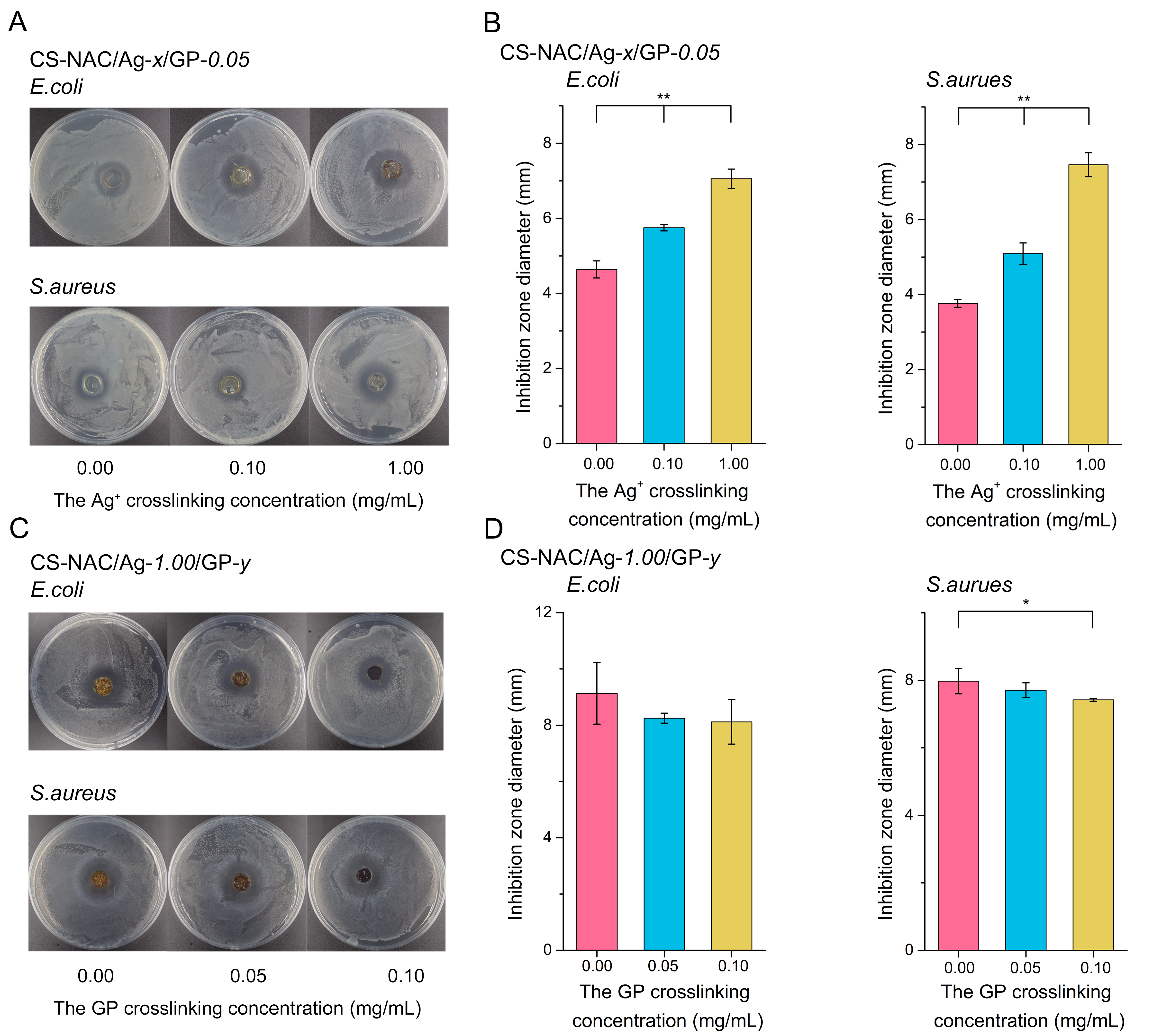
**

**Figure S6** Antibacterial activity of the hydrogels. (A) Photographs of the inhibition zone and (B) statistical data of the zone diameters for the hydrogels with different Ag^+^ concentrations. (C) Photographs of the inhibition zone and (D) statistical data of the zone diameters for the hydrogels with different Ag^+^ genipin concentrations (n = 6, mean ± SD, ***P* < 0.01).


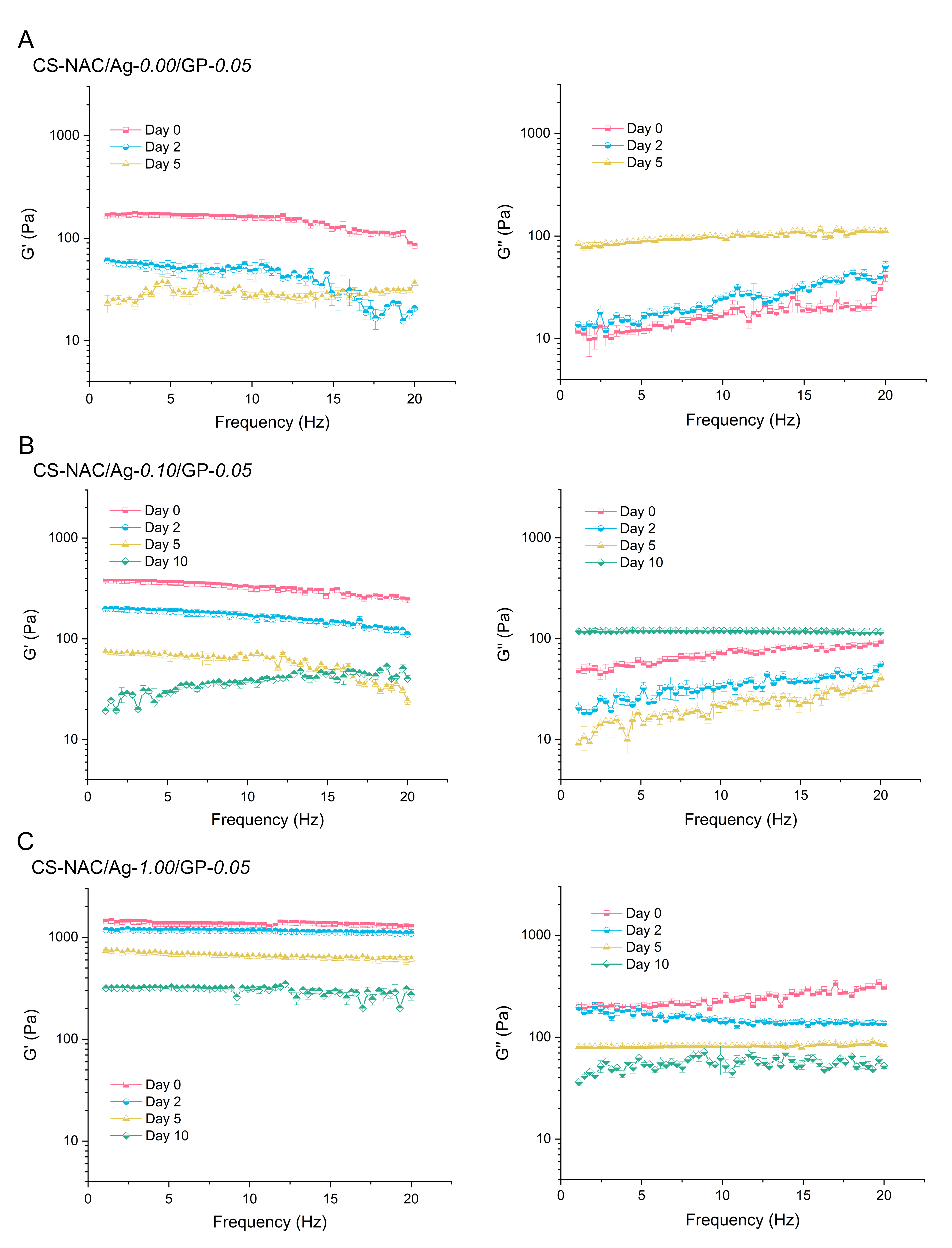


**Figure S7** Frequency sweeps (G′ and G″) of (A) the CS-NAC/Ag^+^-*0.00*/GP*-0.05*, (B) the CS-NAC/Ag^+^-*0.10*/GP*-0.05*, and (C) CS-NAC/Ag^+^-*1.00*/GP*-0.05* on day 0, 2, 5 and 10.
